# Exploring the experiences and coping strategies of international medical students

**DOI:** 10.1186/1472-6920-11-40

**Published:** 2011-06-25

**Authors:** Bunmi S Malau-Aduli

**Affiliations:** 1 Medical Education Unit, School of Medicine, University of Tasmania, 17 Liverpool Street, Hobart, Tasmania, Australia

## Abstract

**Background:**

Few studies have addressed the challenges that international medical students face and there is a dearth of information on the behavioural strategies these students adopt to successfully progress through their academic program in the face of substantial difficulties of language barrier, curriculum overload, financial constraints and assessment tasks that require high proficiency in communication skills.

**Methods:**

This study was designed primarily with the aim of enhancing understanding of the coping strategies, skill perceptions and knowledge of assessment expectations of international students as they progress through the third and fourth years of their medical degree at the School of Medicine, University of Tasmania, Australia.

**Results:**

Survey, focus group discussion and individual interviews revealed that language barriers, communication skills, cultural differences, financial burdens, heavy workloads and discriminatory bottlenecks were key factors that hindered their adaptation to the Australian culture. Quantitative analyses of their examination results showed that there were highly significant (p < 0.001) variations between student performances in multiple choice questions, short answer questions and objective structured clinical examinations (70.3%, 49.7% & 61.7% respectively), indicating existence of communication issues.

**Conclusions:**

Despite the challenges, these students have adopted commendable coping strategies and progressed through the course largely due to their high sense of responsibility towards their family, their focus on the goal of graduating as medical doctors and their support networks. It was concluded that faculty needs to provide both academic and moral support to their international medical students at three major intervention points, namely point of entry, mid way through the course and at the end of the course to enhance their coping skills and academic progression. Finally, appropriate recommendations were made.

## Background

The 2003 changes in the Australian immigration policy have enabled international medical graduates to remain in Australia and obtain access to general medical registration [1]. As a result, the profile of Australian medical schools has seen an unprecedented increase in the number of international students. According to the Australian Education International (2010) [2], the total number of international student enrolments increased by 11.9% from 163,077 in April 2009 to 182,443 in April 2010 with 82% of these enrolments from Asia. The Australian University Medical Education Statistics (2009) [3] reported more than a double increase in the number of international medical graduates from 1999 to 2007.

The large-scale movement of international students into the Australian medical education system means that medical educators need to consider the learning and teaching implications of the increased numbers of international students in university classes. It is crucial that medical educators remain tuned into their diverse students' needs [4] and ensure that these students engage with their academic environment by adopting quality teaching and innovative and inclusive practices. A few studies have addressed the internationalisation of medical education, learning outcomes of international traineeships as well as the learning process of international students [5-8]. However, it has been argued that newer western pedagogic techniques may not be applicable for international students [9].

Over recent years, Australian universities have provided orientation experiences that endeavour to ease the transition of students into the university [10-13]. Some studies have indicated that the difficulties in making a transition to university are not exclusive to international students, and many domestic (including indigenous) students face similar significant transition issues when they arrive at university [10,14]. In coming to university, students are expected to develop new ways of thinking, learning and communicating [15]. However, there are major differences between the challenges faced by domestic and international students (mainly language and cultural issues). Research studies have noted the adjustment issues of this group, particularly its vulnerability to acculturation strains and isolation [16-18]. Others have pointed to a critical need for specific interventions with international students [19,20]. International students are faced with many constraints within their new and unfamiliar cultural and academic domains. These include acculturation, difficulties with English as a second language (ESL), communication and/or learning difficulties, differing expectations of teachers, financial burdens, dealing with isolation, discrimination and other intercultural issues [18,21-23]. Even those international students who arrive from English speaking backgrounds find it difficult to adjust to the Australian accent, talking speed, and the numerous idioms and colloquial language that are employed [9]. International medical students are further inundated with stress right from the start of their medical training as they attempt to adjust to lifestyle changes and increasing challenges incurred by the demands of medical education [24]. Major stress factors for international medical students include curriculum overload, family responsibilities, incurring financial debts, decreased chances for social leisure and inadequate coping skills [25,26].

International medical students are expected to pay a lot more tuition fees, they are also exposed to competency-based assessments which require them to be proficient in communication skills, be able to read the non-verbal cues in an interaction and respond with cultural appropriateness to pass their clinical assessments. Communication proficiency is a core clinical skill in medicine. A physician performs 160,000 to 300, 000 interviews during a lifetime career making the medical interview the most commonly performed procedure in clinical medicine [27]. Evidence-based studies show that effective interpersonal and communication skills are associated with improved health outcomes [28]. Ineffective communication skills are associated with malpractice claims and suits [29] and medication errors [30]. This confirms the need for medical educators to ensure that international medical students are integrated and actively engaged for their successful progression in the medical course.

Research has found that academics are aware of the learning needs of their students, but may be unclear about how best to address those needs [31]. "When considering internationalisation of the curriculum", as academics have themselves claimed, "what is taught should not be separated from how it is taught" ([32], p. [12]). Innovations in classroom practices and in designing assessments are to be found. "Strategies for engaging and extending the full potential of international students by exploring the 'fit' between educational strategies and the possibilities for maximising their potential to succeed in this context are to be devised" ([21], p. [11]).

Studies have shown that both academic and non-academic factors affect the progression of students through higher education [14,33]; however, literature on factors contributing to successful progression of students is limited [34,35]. Given the high tuition fees that international medical students pay, do they feel they are getting value for money? What are their perceptions of their skills, knowledge and expectations, particularly in relation to assessment as they progress through the course? What is the extent to which they demonstrate commonalities and differences with regard to their self-identified coping skills and progression? Answers to these pertinent research questions will provide a better knowledge base for medical educators to understand and respond to the needs of this group of students. Therefore, the study was aimed at exploring the experiences and coping strategies of international medical students at the School of Medicine, University of Tasmania, Australia; to reveal the difficulties and challenges faced and the factors that influenced progression through the medical training program. It is anticipated that the outcomes there from, would help develop better policies and practices which would positively support this group of students.

## Methods

### Participants

Participants were international medical students enrolled in third- and fourth-years of their medical studies at the University of Tasmania. They were from the following seven countries: Malaysia, Singapore, Canada, Hong Kong, Taiwan, Republic of South Korea and Thailand. A total of forty six (46) full fee paying international medical students comprising nineteen (19) third-year students and twenty-seven (27) fourth-year students were identified from the faculty's enrolment database and invited to voluntarily participate in the study. Third- and fourth-year students rotate through different clinical attachments and the training program utilises case-based learning to augment direct patient contact. First and second year students were excluded from the study because they were not involved in clinical attachments and direct patient contact. All the third-year medical students were located in the Hobart city campus, while the fourth-year students were located in one of the three clinical schools in Hobart, Launceston (urban areas) and Burnie (rural area).

### Procedure

Prior to commencement of the study, ethics approval was obtained from the Tasmanian Social Sciences Human Research Ethics Committee. A letter requesting participation in the research project was sent to each prospective participant via email, along with a consent form and a copy of the questionnaire. In order to boost motivation toward response, an information sheet, which included a brief preface outlining the purpose of the research, the criteria for participation, research procedures and participants' rights, was given to each prospective participant. Three weeks after the invitation was sent out, only 10 of the prospective participants responded (six of the third and four of the fourth year students). In order to increase participation and consequently the reliability of the study, further permission was sought and approved by the Ethics Committee to amend the recruitment strategy. The international medical students' representative (third year student) facilitated a face-to-face meeting of all third and fourth year students where the researcher better explained the reasons for conducting the study and the benefits of involvement in the study. Care was taken in describing the benefits of participating so that the students did not feel coerced to participate.

### Materials

This research project included both qualitative and quantitative data collection and analysis as described below:

### Qualitative Data Collection

To best capture the experiences of the students, questionnaire, focus group and personal interviews were used. The primary purpose of the questionnaire was to help structure the interview questions so that they were directly relevant to the students' concerns. The interviews were conducted to explore experiences and perceptions and to gain an in-depth understanding that cannot be obtained from a questionnaire alone. This gave the participants an opportunity to reflect further on their experiences.

#### Questionnaires

Sixteen (16) third-year and ten (10) fourth-year students provided responses to the questionnaire. The first part of the questionnaire focused on the personal details of the respondents, while the second part consisted of eleven open-ended questions (Additional File 1: Appendix 1). The questions were developed from ideas emerging from literature and designed to explore the feelings of acculturation, psychological adjustment and socio-cultural adaptation in relation to cultural and academic adaptation concerns; language and personal support/counselling related issues.

#### Interviews

The interviews were conducted in two stages: (i) two focus group interviews, conducted one week after the questionnaires were administered and (ii) two individual interviews, conducted one week after the focus group interviews. Twelve (eight third-year and four fourth-year) students accepted to participate in the focus group while two (one third-year and one fourth-year) students accepted to participate in the individual interviews. The interviews took place in informal classroom settings, were audio recorded and lasted about 50-60 minutes each. Honesty and confidentiality were emphasised. Specific questions (Additional File 1: Appendix 2) were devised (both for the focus group and individual interviews) from the outcomes of the structured responses given in the questionnaire; this was to ensure that the questions discussed were student-driven rather than research driven. The author conducted the interviews and was not involved in the academic experiences of the students participating in the study.

### Quantitative Data Collection

Participants' 2010 summative examination results were collated and analysed to assess the congruence between perceptions of academic adjustments/clinical experiences and performance in examinations. Performance trends, using different assessment instruments (Multiple Choice Questions - MCQs; Objective Structured Clinical Examinations - OSCEs; and Short Answer Questions - SAQs) were also determined.

### Data Analysis

Emerging themes from the questionnaires were coded and used to develop the interview questions. Using in-depth qualitative analysis; the audiotaped focus group discussions and individual interviews data were collated, independently coded and confirmed by two researchers using pre-developed code lists from the literature and the questionnaire. Illustrative quotes were reported verbatim to support the discussion. Collated examination results for all participants were subjected to statistical analysis using General linear models (GLM) procedure in SAS [36] to compute least square means and their associated standard errors (S.E) and descriptive statistics of the variables. Means of test scores in all exams were computed using the least significant difference technique.

## Results

Sixteen (84%) out of the nineteen third year and ten (33%) out of the twenty seven fourth year international medical students who were invited, volunteered to participate in this study as depicted in Table 1. There were more male than female students. All participants were in the 21-30 years age bracket and 80% of the respondents started the course from year 1. The remaining 20% entered in third year under a bridging program. The sampling population is quite similar to the international student profile at the university, with the larger majority coming from Malaysia [37].

**Table 1:** The profile of participants

Characteristic	Year 3	Year 4	Overall
**Sex**			
**Female**	7	2	9
**Male**	9	8	17

**Age-Group**			
**< 20 years**	0	0	0
**21-30 years**	16	10	26
**31-40 years**	0	0	0
**> 40 years**	0	0	0

**Entry Point**			
**Year 1**	11	9	20
**Year 3**	5	1	6

**Ethnicity**			
**Malaysia**	9	8	17
**Singapore**	4	1	5
**Canada**	1	0	1
**Taiwan**	1	1	2
**Hong Kong**	1	0	1

### Findings

There was an overlap of participants' views in the questionnaire, focus group and personal interviews. Table 2 shows a summary of the questionnaire findings. Participants in both cohorts had similar views and a lot of the issues raised at the focus group discussions resonated in the interview sessions. Data from the focus group and personal interviews were categorised into three major themes namely: Adaptation/cultural issues, academic issues and advice/further suggestions. These three major themes were further subdivided into nine emerging themes as described below and presented with verbatim illustrative quotes from third- (F3) and fourth-year (F4) focus groups; third- (I3) and fourth-year (I4) personal interviews.

**Table 2:** Emerging Themes from the Survey

Cultural Adaptation	
**Factors that aided adaptation**	Senior international medical students, Church and pastoral care, Support from international students office and academic staff, other international students in the class
**Factors that hindered adaptation**	Different lifestyle, English as a second language, Different learning styles, Communication problem, Lack of understanding of the Australian medical health care system, Different foods

**Language Issues**	

**Language problems**	Not used to Australian slangs, Accent problems, limited written, and verbal communication skills
**Communication skills**	Some were satisfied with their level of communication, others indicated that there is room for improvement

**Academic Adjustment**	

**Quality of course**	Satisfied, rated on the average as 7.5 on a scale of 10 points
**Most stressful areas**	Assessment, Objective structured clinical examinations (OSCEs), high expectations
**Most helpful areas**	Peers, clinical attachments, ward rounds, lectures, seniors, bed-side clinical tutorials
**Assessment related issues**	Reflective pieces, oral presentations and OSCEs are stressful, communication constraints

**Personal & Support Concerns**	

**Integration**	Difficulty with Australian culture, cultural shock, rated on the average as 6 on a scale of 10 points
**Coping resources**	Family, friends, peers, seniors, lecturers, church family, holidays, internet access, resilience
**Role of academic staff**	Supportive, approachable, aided better adjustment. However no formal program for adjustment support

## Adaptation/Cultural Issues

### Integration

Most participants felt they had experienced a moderate level of integration into the Australian culture with a rating of 6/10, particularly in the academic arena. Integration had been fostered through the internet, reading books, travelling - 'short trips around Australia during holidays also helps ease the stress and allows for some time off away from Hobart' (F4). Participants who had experienced boarding school in high school felt that they were able to adapt quickly because they were comfortable with living away from home. However, some reported that lack of understanding of the Australian medical health system as well as the objectives and functions of available social services slowed down their adaptation to the Australian culture. Other adaptation-hindering factors that were identified by participants included heavy workload, overwhelming academic demands of the medical course, difficulty in finding accommodation - 'I had some struggle to find house and at the same time I need to attend classes' (F3), difficulty in sourcing for familiar food items (e.g. Halal foods for Muslim students) and the cold Tasmanian weather. Most of the participants expressed experience of discrimination to varying degrees, particularly in the community - 'needless to say that the main issue which troubles me would be discrimination, fortunately not occurring in classes or other teaching places' (F3). Participants reported that there wasn't much help from the school in terms of formal aiding of the integration process.

### Language and Communication Issues

All participants indicated that they spoke and understood English before starting the course. However, most of them expressed that they found it difficult to understand the Australian accent - 'I speak fluent English, the only languages problems were various Australian slang and the Australian accent, prompting me to ask some people to repeat themselves at times' (F4). This problem is exacerbated in class room settings - 'sometimes, when lecturers speak too fast and do not use microphone, I cannot hear what they said in the lectures' (F3). Poor grammar and limited vocabulary to understand and convey messages/information (particularly medical terminologies) were also identified as reasons for language problems - 'I have problem with my grammar. So, it is really too hard to write a 2000-word assignment without making a single mistake at every single line' (F3).

### Support

#### International seniors and peers

Participants indicated that they received the most academic and moral support from senior international students and their international peers. 'Honestly, I think having people who have been there and done that helps a lot, they're able to advise us on things to do and things not to do' (F3). 'We have a common ground and this enables us to share our experiences' (F4). 'Support from senior international medical students were also vital giving us tips and guides on how to get through medical school' *(I3)*. Peers from the same country of origin and other international students also provided support and formidable friendships - '... I also have weekly dinners with two other international friends' (F3).

#### Domestic peers

A few participants indicated that staying with Australian students made the transition easier - 'staying with Australian students has definitely helped me adapt' (I4). However, most participants stated that there was obvious segregation between international and domestic students, with very little or no support at all from their domestic peers 'although this may not be intentional' (F3). 'Making friends with local Australians was not an easy task as either they already had their own click of friends and were unsure of who we were (i.e. some thought we were repeating the year and so on) or they simply were not interested in making Asian friends as they seem to think we had a very poor command of the English language' (F3). Participants indicated that they scarcely mingle with their domestic peers because social activities for the latter group involved high consumption of alcohol. 'I am able to hold conversations but depth of relationships does not go any further than inside the classroom' (F4).

#### Staff

Participants acknowledged academic support to varying degrees. While some indicated that they had little need for help, others reported that most of the academic staff members were extremely helpful and approachable. In their times of need, the students felt very much supported and were able to 'stand on their feet again'. 'I am happy and grateful for the support given me in my trying times' (I4). Others stated that as full fee paying students, they should be getting more lecturer-student interface sessions to maximise learning in both lecture and clinical settings. 'It would be better if they spend time to understand my previous background and experience' (F4). 'I always feel that my classmates are very smart and they can learn things very fast. For me, I have to take more time and do more practices to learn these things. Sometimes, I feel that it is hard to follow them' (F3). They noted that although there was some amount of support services and programs organised by the International Student Office, but 'sometimes it is difficult to identify the right person to talk to about academic/personal issue, also heavy workloads and logistics of location did not permit us to attend such useful programs' (F3).

#### Family

Family members provided participants with a great deal of moral and financial support. 'Family is very important as well but since they are so far away, there really isn't much they can do apart from encouraging us I guess' (F4). 'I call home once a week as I can make cheap overseas calls to my family and friends (I4).

### Coping Resources

The most significant factor that has aided participants in their persistence and progression in the medical program is their key goal of becoming medical doctors (both the humanitarian side of it and the prestige that comes with the profession). - 'I think just being really resilient and independent helps. Yes there are times when you feel like giving up but knowing what the end goal is helps a lot' (I4). Other motivating factors identified included encouragement and advice from family and senior international medical students, pressure to perform academically due to high tuition fees, regular meetings with peers (other international medical students) to read, cook, practise OSCEs and share experiences. Participants indicated that they had at some stage in their pursuit of the medical degree, considered withdrawing, and this was due to the challenges of the medical education (stress and workload). However 'withdrawal isn't considered as an option because of the shame and unhappiness it would cause my family members' (I3). Participants rather concentrated on the positive outcomes of the process and the prestige attached to completing the medical degree.

### Financial issues

Participants indicated that because of their high financial commitment to the medical program, they were constantly under pressure to perform well so as not to let their families down - 'failing and repeating a year in the medical school causes great emotional and financial distress' (I4). Participants expressed desire for the school, faculty and university at large to be more proactive in providing formal moral support to help during such periods, 'I believe that it is the responsibility of the school to advocate and to look after the affairs of all their students' (F4). 'In the Australian culture, one is expected to call for help if needed', but we come from backgrounds where it is okay to offer people assistance without them asking for it' (F3).

## Academic Issues

### Quality of the MBBS Program

Participants were quite impressed with the quality of the medical program, the teaching structure and academic support provided by the school - 'I am very satisfied with the quality of the program and academic support available. I would rate it a 7.5/10, with a few aspects needing improvement and coordination' (F4). Participants were quite pleased with the integrated system of learning which offered them early exposure to clinical scenarios from year 1. They indicated that the school's model of case-based and self-directed learning allowed them to gain a lot of confidence and encouraged them to gradually adopt active learning strategies. They highlighted the need for additional tutorials for international students to help with 'brushing up on communications skills' (F3), they felt tutorial groups set up for only international students will help them to be more confident to ask questions.

### Learning styles

Most of the participants indicated that they have come from conservative backgrounds where the teacher can't be questioned and rote learning is prevalent. Therefore, 'adjusting to a self-inquiry learning style that requires lateral thinking has been very difficult' *(I4)*. They have had to change the way they prepare for examinations and some indicated that this realisation came for them only after they had failed one or more exams. With the majority of the international students coming from Asia, participants expressed disappointment at 'the lack of assistance from the school in explaining the expectations and the western learning style' (F3). Some participants explained that they used their 'old' learning style for written exams and adopted the 'western learning style' for OSCEs (F3).

### Assessment

One major issue that resonated from both year groups was the difficulty international students experienced with OSCEs - 'the OSCE examination is by far the most stressful assessment' (F3). Many international students found it difficult to ascertain what was required and expected for OSCEs, - 'students need to be taught about the cultural expectations in relation to communication skills' (F4). Oral presentations, reflective pieces, essays and written assignments were also seen as stressful 'as we often got stuck with word choices and need to translate the meaning of words in our heads before expressing them, an arduous time consuming task' (F3).

**Figure 1:**
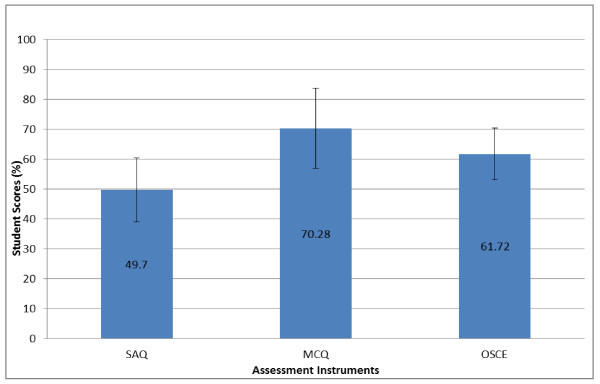
**Performance of students using three different assessment instruments**.

**Figure 2:**
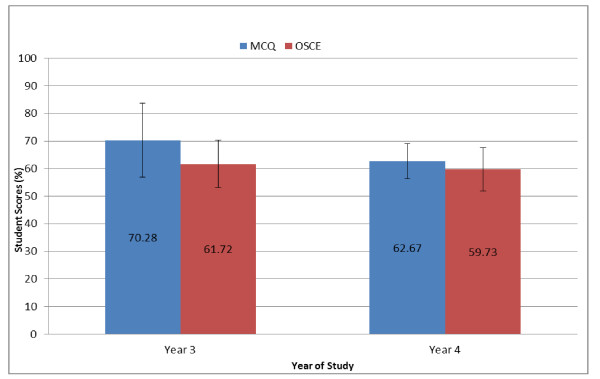
**Performance trends in year 3 & year 4 students' exam scores**.

Quantitative analysis of the first and second semester examination results indicated high congruence between students' perceptions of the difficulty level of the various assessment instruments and their actual performance in the examinations. Students obtained significantly higher ((p < 0.001) scores in the MCQ exams (70.29%) compared to the OSCEs (61.72%) and the SAQs (49.71%) as shown in Figure 1. A similar pattern was observed in the performance of both cohorts (Figure 2), indicative of verbal and written communication problems. There was improved students' performance in the second semester OSCE compared to the first semester (data not shown). Generally, participants felt it would be better if they received more specific and meaningful feedback on their assessments.

### Advice and Suggestions

Participants gave the following advice to first year international students:

• *'Study hard, be hopeful and don't give up' (I4);*

• *'Speak loudly, clearly and confidently, and maintain good eye contact as that is the culture here, unlike back home where you are not allowed to question your lecturers' opinions or even make eye contacts with them' (I3);*

• *'Be open and don't be judgmental, respect people even if you don't agree with their views' (I3);*

• *'The learning style and academic focus are different, with more of rote learning and focus on acute diseases back at home, while the Australian learning style involves application and self inquiry with a focus on chronic diseases' (I4);*

• *Seek for advice from senior international medical students but also integrate with the domestics so as to adjust quickly to the Australian culture and lifestyle' (I4)*.

Participants suggested that the school would need to be more proactive in aiding the adaptation and integration of international medical students into their new learning environment indicating that 'having paid a lot of money for the course', they felt they 'were entitled to more support services from the school and university' (F3) 'with the setup of formal international medical students' association to better convey any issues or concerns' that they might be facing *(I3)*. Participants indicated that there were three crucial points in their academic journey where there was need for the school to provide both moral and academic support, namely at the: point of entry (1^st ^year), mid-way - i.e. during clinical transition (3^rd ^year) and point of exit (5^th ^year).

## Discussion

This study has shown a lot of commonalities in the diverse students' narratives as it explored their coping strategies and academic progression in the face of prevailing adaptation challenges. It has also confirmed early research findings that international students are constrained by acculturation, socio-cultural adaptation, language, and learning/communication difficulties [25,26]. This study has also indicated that the degree of difficulty in adjustment to host institution varies from student to student. The greater the similarity between the characteristics of students and the new environment, the easier the interaction as acculturation, satisfaction and achievement of the host country's expectations enhances adjustment [16]. Students who have come from backgrounds with English as the first language felt they were able to adapt faster to the Australian culture and were able to make satisfactory social interactions with their domestic peers and people in the community, while those who felt there were a lot of differences between their culture and the Australian culture were more introverted.

Another major area of concern for international medical students is language issues, particularly as proficiency in verbal and non-verbal communication skills is of high importance in the medical degree. Host language proficiency is considered an important variable in determining successful cross-cultural adaptation. Empirical studies have reported that competence in the host country's language is at the centre of the acculturation process and successful communication is necessary to feelings of psychological well-being and satisfaction in life [17]. Pearson-Evans (cited in Mehdizadeh and Scott, 2005, [[38], p. [7]], in a study of Irish students in Japan reported that: "linguistic skills posed one level of difficulty, but interpreting non-verbal behaviour and the underlying communication rules, based on cultural values and cultural logic, were the most challenging problems they faced". This study confirms earlier studies that language difficulties can serve as a communication barrier for international medical students [18,21,39]. Participants expressed the fact that they could not interact effectively with their domestic peers because they lacked discussion topics and due to their different social lifestyles. They also alluded to the fact that they had difficulty in understanding the heavy Australian accent of the lecturers. They also felt stressed about examinations particularly because of their grammar and communication deficiencies.

### Given the high fees that international medical students pay, do they feel they are getting value for money?

Although the participants felt the medical program was of high quality, they indicated that the support provided by faculty to international medical students was very little and needed a lot of improvement. This echoes the report of Sherry *et al*., (2003) [40], that there is a high correlation between student satisfaction and perceived level of support received. Participants clearly indicated that the support provided to them by the university was inadequate as they were unable to attend support programs organised by the international office. However, they have looked inwards and found their own support networks in seniors, peers for emotional and academic support, and family members for financial and emotional support. Major (2005) [41] reported that the learning environment plays a central role in promoting persistence in students as it provides catalysts for frequent and consistent interaction in formal and informal settings which results in active engagement with the learning process. Participants had varying levels of engagement with their learning environment, those who interacted more with their domestic peers and lecturers found it less daunting to seek for assistance and felt they were progressing well with their academics.

### What are the perceptions of their skills, knowledge and expectations, particularly in relation to assessment as they progress through the course?

In this study, the perceptions of the students in relation to their skills and knowledge was highly reflected in their performance in the various assessments as they performed better in the MCQ exams than in the OSCEs and the SAQs. This may be indicative of a learning gap in the application of basic science knowledge to clinical context. It may also point to the existence of some communication issues as language is the vehicle of communication and weakness in language can act as a barrier to learning [42]. Studies conducted on the poor performance of international medical graduates (IMGs) reported that establishing rapport with patients and responding to their emotions was very challenging for the IMGs because they did not know how to express empathy in a different culture [43]. International medical students may have similar communication deficiencies because they may have problems with question formation, understanding informal colloquial language and negotiating treatment plans. The observed improved students' performance in the second semester OSCE suggests that assessment drives learning as poor performance in the first semester elicited a change of learning style. The poor performance of the third year students in the SAQ exams confirms the report by Campbell and Li (2008) [39] that international students had difficulties expressing their opinions in writing due to insufficient knowledge of academic conventions. This confirms that there is the need for host institutions to provide international students with extra support on their knowledge of academic conventions and their communication skills to better integrate them into their new learning environment.

### What is the extent to which they demonstrate commonalities and differences with regard to their self-identified coping strategies and progression?

Despite all the challenges faced by the participants, they demonstrated their persistence to carry on with the course rather than choosing to opt out. This is largely due to their high sense of responsibility towards their family, their unflinching focus on the goal of graduating as medical doctors and support networks from senior international medical students and international peers. This study underscores the report by Sparrow *et al*., (2009) [44], that the setting of personal goals motivates students to persist with their studies and in many cases, gives them the resilience to overcome barriers to academic success. Support is well recognised as an important retention factor for first year students and particularly peer support [13]. Participants indicated that they set goals for themselves as a group and reward whoever gets the best results in examinations. This has added a dynamic component to their learning experience.

### Limitations of the Study

This study is limited by the participation of only third- and fourth-year international medical students. Obtaining perspectives from the first- and second-year international students may elicit different types of challenges experienced in the early years of the course and resilience/coping strategies devised by this group. Furthermore, the use of only volunteers does not allow one to capture the views of all members of the cohort and some of the responses may have been biased due to the overrepresentation/underrepresentation of a particular group. Another limitation was that using assessment results for only one year of study may not be sufficient to make generalised statements about the performance of international medical students across different assessment instruments. Finally, the views of the academic staff and domestic peers could have been captured too to understand their challenges and possible benefits of international medical education from their perspectives.

### Implications for Practice

This study confirms that international medical students have to deal with issues such as cultural adaptation, language barriers, academic and financial constraints. It also reveals that they employ efficient coping strategies such as seeking and maintaining strong support networks, setting and striving to achieve high academic goals for themselves and endeavouring to actively engage themselves in their learning environments. They use all these strategies to organise their lives, persist, overcome challenges and succeed academically. This understanding highlights for medical educators, the relevance of students' goals to their academic, personal, emotional and professional growth. Strong reliance on support networks indicate that faculty can provide international medical students with both assessment-linked and social network opportunities that will foster better integration with their domestic peers. Learning environments that are designed to be interactive and challenging, and are compatible with students' goals, enhance individual learning and also aid the development of formal and informal communities that create a sense of belonging. Medical schools need to ensure that lecturers and clinicians take cognisance of the communication issues that these students face and therefore equip them with the required skills. Also, discussions on differences in the perceptions of the lecturer-student role need to be explored as well as cultural differences that impinge on learning styles, communication skills, patient-centred care and professionalism. This emphasises the need for medical schools and higher education (HE) institutions as a whole to realign their pedagogical practices to suit the needs of both domestic and international students.

## Recommendations

The participants have suggested that three stages of interventions need to be initiated and the recommendations derived from the study include the following:

### General recommendations

• Pre-arrival information packages/kits with tips on the weather, dominant culture and lifestyle of the host country/institution should be organised and sent to new international students.

• Given that international medical students have a heavy workload and are constrained for time to attend support programs organised by the International Office, it will be of utmost benefit if international student support programs are set up within the school. These should be under the auspices of an academic coordinator so that relevant activities can be organised within suitable time frames to maximise attendance.

• Support programs should be organised between the first and third week of a new academic session and in between semesters before academic pressures begin to mount.

• To alleviate issues around lack of social interaction between domestic and international students, regular fun activities (excluding drinking due to religious and cultural beliefs of the international students) such as sports, tours and cooking competitions can be organised. High level involvement of the medical students' union is recommended to foster these interactions.

• Academic interactions between international and domestic students can be fostered by initiating activities that utilise the cultural diversity of the student cohort to develop the necessary clinical skills in both groups. As much as international students need to learn to empathise and communicate across cultures, so do domestic students need to learn to communicate quite complex medical information to patients with poor English skills. Use of diverse groups of patients/role-players from different cultural backgrounds for OSCEs will benefit both groups immensely.

### Point of entry interventions

• Providing a compulsory 2-5 day bridging/orientation program for first year international students (prior to resumption date for whole cohort), where staff of the International Office and other support organisations within the community can provide the students with relevant information/package on available resources will be highly beneficial.

• Orientation activities can include guided tours, presentations on important academic requirements, colloquial language and useful tips for daily survival. Enlisting the support of seniors (domestic and international) in small group discussions in relation to expectations, cultural differences and expected academic standards may foster faster integration.

### Mid-way interventions

• Tutorials/workshops can be organised to orientate international students on the host country's health care system

• Training sessions on confronting, challenging and sensitive clinical examinations, as well as appropriate clinical and colloquial language can be provided. Engaging senior students (to share their experiences) can be reassuring.

• In situations where new international students join the cohort mid-way through the course, organising practice OSCE sessions as well as mini orientation programs with guided tours of the library, hospital and other services will be useful.

• Organising workshops where clinicians who have worked overseas can share their experiences with the cohort may create awareness and facilitate discussions around cultural differences in the health care system. This will encourage interaction between domestic and international students.

### End of course interventions

• Usually, at the end of the course, how international graduates settle back into their home countries and learn the ropes of working in their own medical system is rarely considered by the host institution. Providing information sessions/support programs at this stage on the challenges of reverse culture shock might help alleviate some of their concerns and equip them for faster integration into their home country's health care system.

## Conclusions

This study provides an in-depth understanding of the challenges international medical students encounter, and the factors that influence their progression through the medical training program. Although the majority of international medical students pursue their academic goals with strong determination, academics and host institutions still need to play a major role in supporting them with the management of challenges in their cultural academic adaptations. It is important that lecturers review their pedagogical practices and host institutions need to develop programs that aim to mutually benefit lecturers, domestic students and the international students. Finally, in order to enhance integration and academic progression amongst international medical students, faculty needs to provide both academic and moral support to their international medical students at three major intervention points, namely point of entry, mid way through the course and at the end of the course.

## Competing interests

The author declares that they have no competing interests.

## Authors' contributions

BMA conceived the study, coordinated the project, analysed and interpreted the data and developed the manuscript.

## Author's information

BMA: BSc (Hons), MSc, PhD, is a Lecturer in Medical Education (Assessment) at the School of Medicine, University of Tasmania. Her research areas include the use of quality assurance processes to improve learning and teaching in higher education and the design and analysis of assessment instruments.

## Pre-publication history

The pre-publication history for this paper can be accessed here:

http://www.biomedcentral.com/1472-6920/11/40/prepub

## Supplementary Material

Additional file 1 **Appendix**. Contains Appendix 1 and 2, Questionnaire and Interview Questions. Click here for file
