# Supplementary material for: Exploring the experiences and coping strategies of international medical students
Source: BMC Med Educ. 2011 Jun 25;11:40. doi: 10.1186/1472-6920-11-40 (PMC3141796; doi:10.1186/1472-6920-11-40)
Supplement: Additional file 1 — Appendix. Contains Appendix 1 and 2, Questionnaire and Interview Questions. [file 1472-6920-11-40-S1.DOC]

## Appendix 1: Questionnaire

Please read and consider each of the following questions carefully. Your candid responses are essential to ensure the reliability of this study. Please ensure that you answer all the questions.

Part A: Personal Details

| Surname |  |
| --- | --- |
| Given Names |  |
| ID Number |  |
| Year of Study |  |
| Country of Origin |  |
| Gender | Male  Female |
| Age-Group | < 20yrs  21-30 yrs  30-40 yrs  > 40 yrs |

**Part B:**

**Cultural Adaptation**

- Comment on the various aspects that have aided you in adapting to your university life as an international medical student.
- Comment on the various aspects that have hindered you in adapting to your university life as an international medical student.

**Language Issues**

- Identify areas (if any) in which you encounter language problems
- Comment on your communication skills

**Academic Adjustment**

- Comment on your general satisfaction with the quality of the course
- What part of your academic life is most stressful?
- What part of your academic life is most helpful?
- What is your current experience of the role of the academic staff in the adjustment process?
- Comment on stress generated by the different assessment instruments you are exposed to

**Personal and Support Concerns**

- To what extent have you experienced satisfactory integration into the Australian culture?
- What resources do you use as strategies for coping?

## Appendix 2: Interview Questions

1. You have succeeded in your studies to reach this stage in your academic career. Please reflect on your persistence and identify significant factors that you think have helped you reach this point in your study?
2. Have you ever considered withdrawing from the course? If yes, give reasons for considering withdrawal. If no, give reasons for not withdrawing.
3. Have any serious issues affected your studies and how have you coped with them? (Probe for institutional support and gaps)
4. If you were speaking to first year international medical students, what advice would you give them about successful progression in the medical degree?
5. Is there anything we have not covered that you would like to comment on?
